# The smokeless tobacco habit and DNA damage: A systematic review and meta-analysis

**DOI:** 10.4317/medoral.22846

**Published:** 2019-03

**Authors:** Juliana-Larocca de Geus, Letícia-Maíra Wambier, Alessandro-Dourado Loguercio, Alessandra Reis

**Affiliations:** 1 Professor. Department of Dentistry. Paulo Picanço School of Dentistry, Fortaleza, CE, Brazil; 2 Professor. Department of Dentistry. Guairacá Faculty, Guarapuava, PR, Brazil; 3 Professor. Department of Dentistry. Positivo University, Curitiba, PR, Brazil; 4 Professor. Department of Dentistry. State University of Ponta Grossa, Ponta Grossa, PR, Brazil

## Abstract

**Background:**

The aim of this systematic review was to evaluate the frequency of micronuclei or other DNA damage in the oral mucosa of adults that have smokeless tobacco habits compared to adults that not have these habits.

**Material and Methods:**

We searched PubMed, Scopus, Web of Science, LILACS, BBO and Cochrane Library and SIGLE. We also surveyed gray literature. We included only clinical trials that compare the frequency of micronuclei or other DNA damage in the oral mucosa of adults that have smokeless tobacco habits compared to adults that not have these habits. Quality assessments of the selected trials were evaluated by two independent reviewers, using the Effective Public Health Practice Project – (EPHPP) with modifications.

**Results:**

After the database screening and removal of duplicates, 2574 studies were identified. After title screening, 172 studies remained, and this number was reduced to 25 after careful examination of the abstracts. The standardized mean difference of the frequency of micronuclei between groups was 1.88, with a 95% confidence interval of 1.40 to 2.36 (*p*< 0.00001). In all analyses heterogeneity was detected.

**Conclusions:**

Despite the heterogeneity of studies, the frequency of micronuclei was significant bigger in adults who have the smokeless tobacco habit when compared to those not have this habit. The same occurred with the frequency of binucleated cells, karyolisis and karyorrhexis.

** Key words:**Micronucleus tests, Tobacco, smokeless, DNA damage.

## Introduction

Tobacco use has been a major component of life style factors and there are a variety of ways tobacco is consumed (1). Apart from smoke tobacco, the smokeless tobacco use has been associated with several abnormalities of the oral mucosa, such as oral cancer (2).

Snuff is a smokeless tobacco made from ground or pulverized tobacco leaves, which is inhaled into the nasal cavity, delivering a swift hit of nicotine and a lasting flavoured scent (especially if flavouring has been blended with the tobacco) (3). Chewing tobacco is a type of smokeless tobacco product consumed by placing a portion of the tobacco between the cheek and gum or upper lip teeth and chewing (3).

Chewing tobacco is typically manufactured as several variety of products, such betel quid, which contains areca nut and a variety of ingredients, including betel leaf and tobacco (4); sadagura, which contains sun dried and roasted tobacco leaves along with very small amount of fenugreek seed and aniseed for flavor; or “maras powder”, made from the leaves of Nicotiana Rustica L., which is dried, powdered and then mixed with ashes of wood from oak, walnut and grapevine (5). In addition to these substances, other products can be mixed and used as smokeless tobacco habits, such tamol, mava, lime (6), khani with toothpaste (7) etc.

Studies have revealed high potential carcinogens in snuff and chewing tobacco. The DNA damage in the cells from oral mucosa of tobacco chewers or snuff users usually sign the genotoxicity potential of the smokeless tobacco habit (8). One of the best techniques for studying the effects of environmental factors on genetic stability in human cells is the micronucleus test (9). A micronuclei (MN) is formed during the mitotic division of the cell. It contains full or acentric chromosome fragments, which are not included in the main nucleus. Increased MN number is regarded as an indirect indicator of quantitative and structural chromosomal disorders of cells that may be caused by various agents and is considered an early marker of carcinogenesis (10).

Other chromosomal aberrations, such as pycnosis, binucleated cells, anucleated cells, are excellent biomarkers of exposure to the chromosome-damaging agents in tobacco (11). For the evaluation of MN or other DNA damage different staining methods can be used, such Feulgen (11), Giemsa (12) or Papanicolau (13).

It has been demonstrated in a recent systematic review (14) that smoking promotes a higher frequency of MN compared to non-smokers. Regarding smokeless tobacco, most of the recent studies have shown that the frequency of MN is significantly higher in chewing tobacco and snuff users than in non-users (1,8,11,15), but there is still a controversy in the literature (16-18).

In face of the controversial results published in the literature, the aim of this systematic review of the literature was to answer the following PECO question (P – patient; E – exposure; C – comparator; O – outcome): Does the smokeless tobacco habit increase the MN frequency or DNA damage in the oral mucosa of adults compared to adults that do not have this habit?

## Material and Methods

The methodology we describe here follows the sequence from a previously published systematic review and meta-analysis from our research group (14). In both articles, we followed the Preferred Reporting Items for Systematic Reviews and Meta-Analyses statement (19).

-Protocol and registration

This study was performed from July to November 2017 at the State University of Ponta Grossa, Paraná, Brazil. The study was registered at the International Prospective register of systematic reviews (PROSPERO CRD42015032354) available at https://www.crd.york.ac.uk/PROSPERO/.

-Information sources and search strategy

For the Pubmed database, we developed the search strategy based on the concepts of patient and intervention from the PECO question described at the end of the introduction section. Within each concept, we combined the controlled vocabulary (Medical Subject Headings [MESH] terms) and free keywords with the Boolean operators “OR”. Then, the concepts were combined with the Boolean operator “AND” to restrict the search. The outcomes evaluated were the frequency of MN or other types of DNA damage (anucleated cells, binucleated cells, pycnosis, karyolisis, karyorrhexis, chromosomal aberrations, nuclear buds and broken eggs).

The Pubmed search strategy was adapted for the other electronic databases. The reference lists of all primary studies were hand-searched for additional relevant publications as well as links with related articles of each primary study in the PubMed database. No restrictions on publication date or languages were imposed in the search strategy.

We also inspected the gray literature by looking up abstracts from the International Association for Dental Research and their regional divisions (1990-2016), the System for Information on Grey literature in Europe (SIGLE), dissertations and theses using the ProQuest database, as well as the Periodicos Capes Theses database. We searched for ongoing trials in clinical trials registries (Current Controlled Trials (www.controlled-trials.com), International Clinical trials registry platform (http://apps.who.int/trialsearch/), the ClinicalTrials.gov (www.clinicaltrials.gov), Rebec [www.rebec.gov.br), and EU Clinical Trials Register [https://www.clinicaltrialsregister.eu)).

-Eligibility criteria

We included cross-sectional clinical trials that compared the frequency of micronuclei or other DNA damage in adults with and without smokeless tobacco habits. Clinical studies were excluded if they: 1) did not evaluate the micronuclei frequency or any other type of DNA damage specified above; 2) were conducted in children.

-Study selection and data collection process

After database screening, we removed duplicates and selected possible eligible articles according to title and abstracts. Two of us (L.M.W., J.L.G.) obtained full-text articles and classified them according to the eligibility criteria. We used pilot-tested, customized extraction forms to register details about the studies, such as study design, participants, interventions and outcomes. We gave each study an identification number (study ID), combining the first author name and the publication year.

-Risk of bias in individual studies

The risk of bias of the selected trials was evaluated by two independent reviewers (J.L.G. and L.M.W.), using the modified scale Effective Public Health Practice Project (EPHPP) (20). This scale contains the following components: sample selection, study design, identification and treatment of confounders, blinding of outcome assessors and of participants, reliability and validity of data collection methods, and withdraws and dropouts. According to a standardized dictionary, these components were rated as strong, moderate, or weak.

The overall rating for the study is determined by assessing the six component ratings. Studies are considered strong if they did not have any weak ratings and had at least four strong ratings. Studies with less than four strong ratings and one weak rating were considered moderate. Finally, studies with two or more weak ratings were considered weak.

As in our review we only included cross-sectional clinical studies, the study design item was not considered in the quality assessment. Additionally, participant’s blinding is not important for this type of study, so only the assessor’s blinding (who evaluate the samples at microscopy) was considered in the evaluation.

-Summary measures and synthesis of the results

We merged data for meta-analysis when: 1) more than one staining method were used to assess the frequency of micronuclei, 2) different areas were used for smear collection of exfoliated cells, and 3) when more than one smokeless tobacco habit was included in the eligible study.

Data were analyzed using Revman 5 (Review Manager version 5.2, Cochrane Collaboration, Copenhagen, Denmark). Continuous data were collected from eligible studies as the mean frequency of micronuclei or other DNA damage. The random-effects models were employed for the continuous data. Only studies classified as strong and moderate were meta-analyzed.

## Results

-Study selection

**Figure 1:**
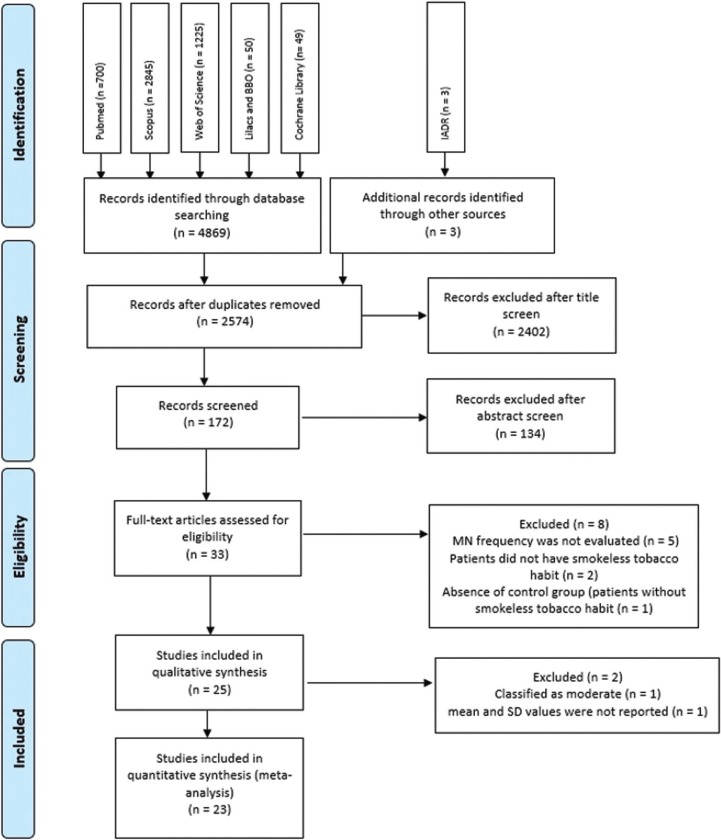
Flow diagram of study identification.

After database screening and removal of duplicates, 2574 studies were identified (Fig. 1). After title screening, 172 studies remained, and this number was reduced to 25 after careful examination of the abstracts.

-Characteristics of included articles

The characteristics of the 25 studies selected are listed in  Table 1,  Table 1 continue,  Table 1 continue-1. The type of smokeless tobacco habit included tobacco chewers (8,15,21-25) and snuff users (26,27). Different tobacco mixtures were evaluated in the 25 studies. The majority of studies evaluated maras powder (13,16,17) and betel quid (1,12,28).

**Table 1:**
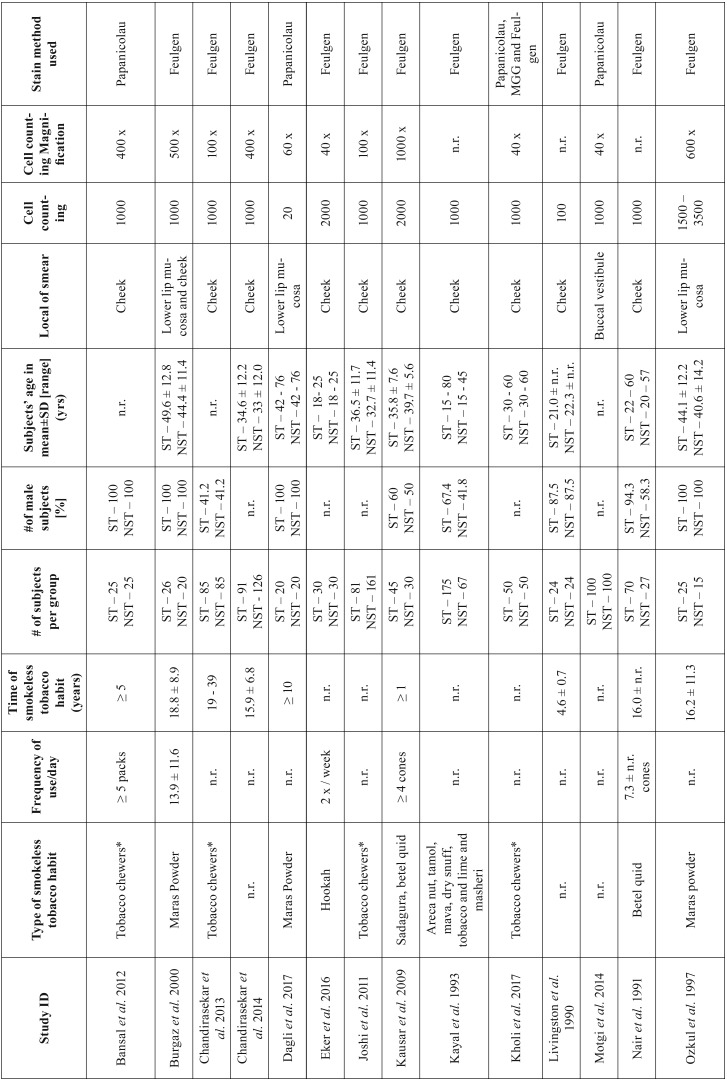
Summary of the studies selected for this systematic review.

**Table 1 continue:**
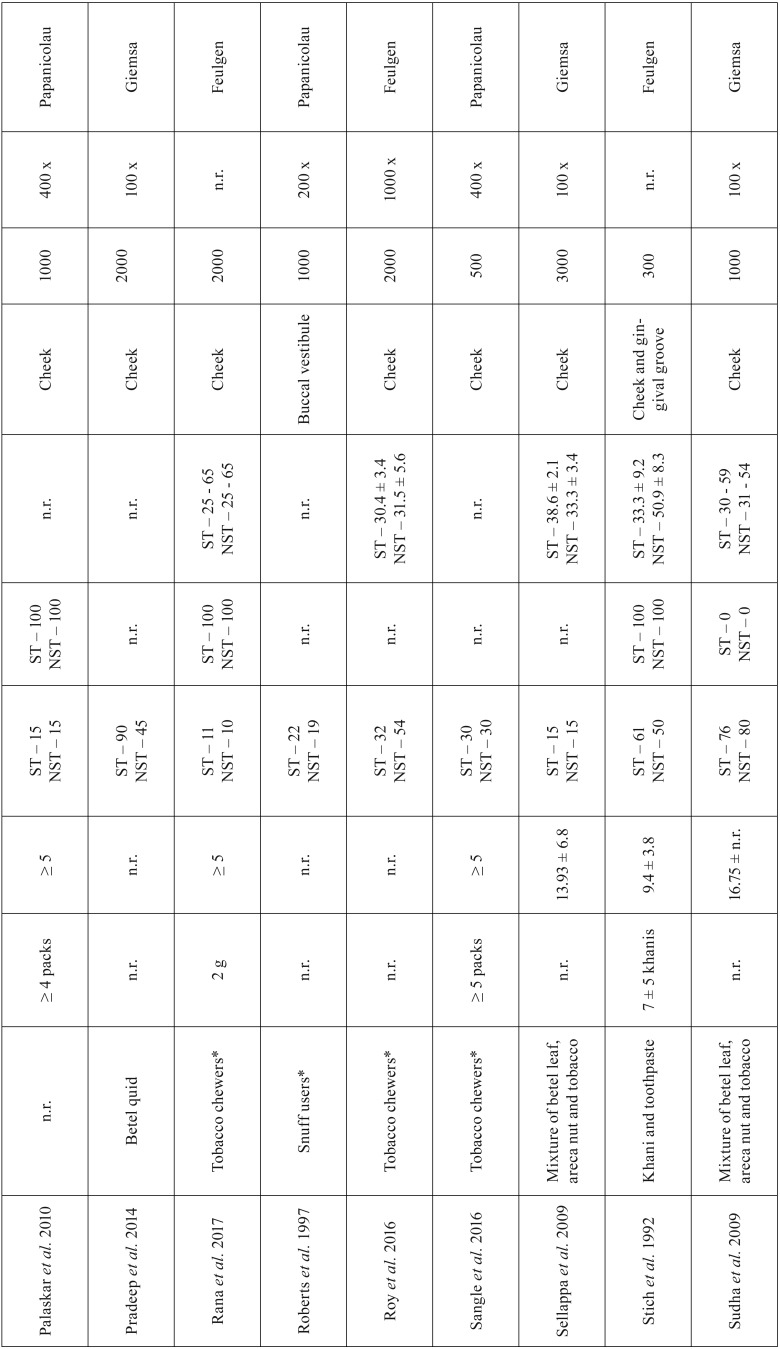
Summary of the studies selected for this systematic review.

**Table 1 continue-1:**
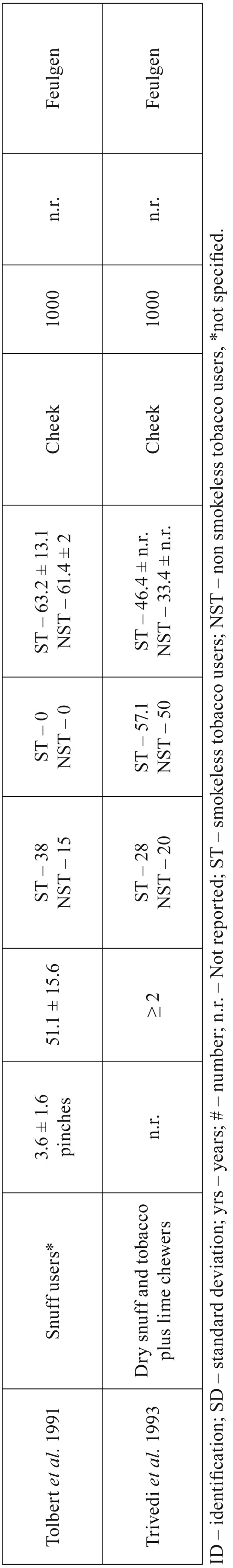
Summary of the studies selected for this systematic review.

The frequency of smokeless tobacco use a day was variable and measured in different units of measurement, but most of the studies did not report this information (6,8,11-13,16,18,21,22,24,26).

The mean use of smokeless tobacco habit was up to 51 years (1,7,11,13,15-18,21,23,25,27). The smallest number of participants per group was ten (23) and the highest was 175 (6). In the studies that report the gender of participants, most of them were male (1,6,7,13,15-17,23,28,29,32), with exception of three studies (21,27,31). The range of participants age varied from 18 to 80 (1,6-8,11,13,16-18,22-24,27).

According to the local of smear collection of exfoliated cells, most of studies (n = 19) used the cheek mucosa (1,6,8,11,12,15,18,21-25,27). The number of cells counting per participant ranged from 20 to 3500, with the great majority (14 out from 25 studies) counting on 1000 cells (6,8,11,15,17,21,22,26-28,30). In relation to the magnification used to count the cells, it varied from 40 x (22,30,34) to 1000 x (1,24).

According to the staining technique used in the cytologic smears, the Feulgen stain was the most used method applied in fifteen out of the 25 studies (1,6-8,11,16,17,21,23,24,27).

Other genotoxicity parameters were evaluated in some studies: nuclear buds, binucleated cells, anucleated cells, karyolysis, karyorrhexis, condensed chromatin, chromosomal aberrations and pycnosis.

Assessment of the risk of bias 

The selected studies quality assessment is presented in  Table 2. Few full-text studies reported adequately items to allow evaluation of the selection bias, confounders, blinding, data collection methods, withdrawals and dropouts. In summary, from the 25 studies, seven were considered strong (7,8,13,23,31,33,34), one was considered weak (26) and the majority of them (sixteen studies) were considered to have a moderate quality (1,6,11,12,15-18,21,22,24,27).

**Table 2:**
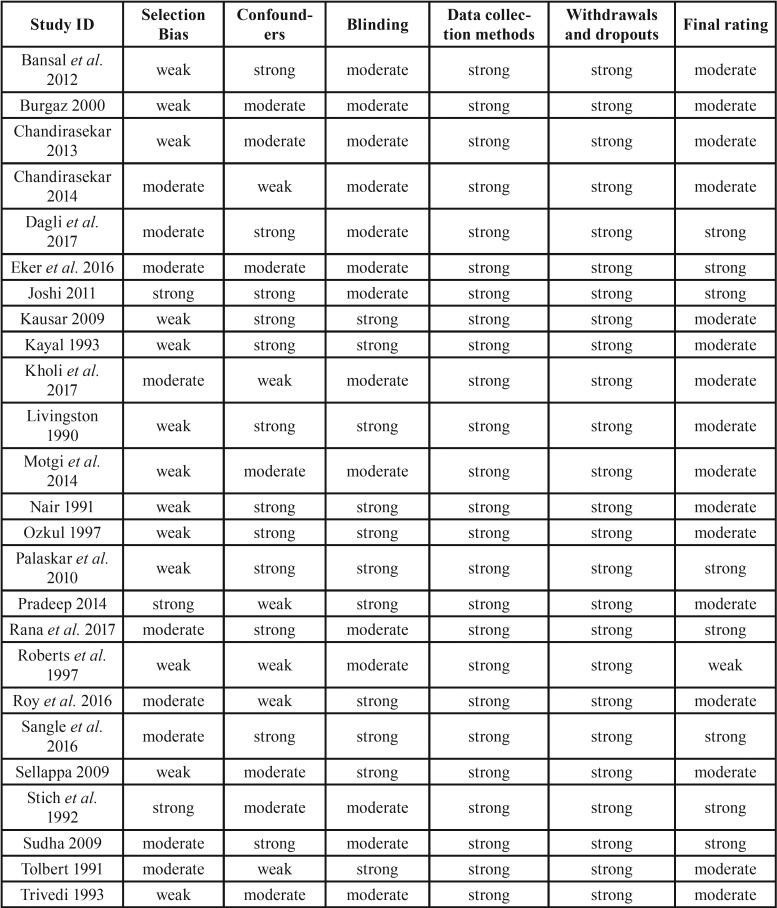
Quality assessment components and final rating of the studies.

-Meta-analysis

All meta-analysis was performed on studies classified as strong and moderate, a total of 24 out of the 25 studies. From these 24 studies, one did not report the mean and standard deviation values, preventing us from including it in the meta-analysis of the present study (13).

Frequency of micronuclei

**Figure 2:**
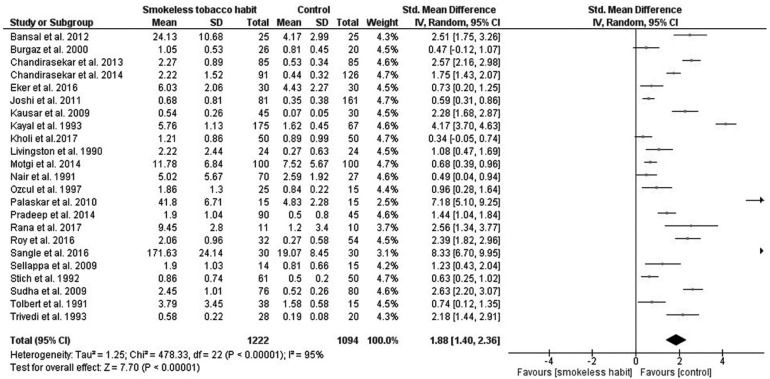
Forest plot of the frequency of micronuclei for smokeless tobacco habit vs control.

This analysis was based on 23 studies (1,6-8,11,12,15-18,21-25,27). A significant higher MN frequency were identified in smokeless tobacco users (standardized mean difference (SMD) = 1.88, with a 95% confidence interval (95% CI) varying from 1.40 to 2.36 (*p* < 0.00001; Fig. 2). Data were heterogeneous (chi-square test, *p* < 0.00001; I2 = 95%; Fig. 2), which means that all studies included in the analysis did not share a common effect size.

-Frequency of binucleated cells

This analysis was based on 5 studies (1,8,24,27,34). A significant higher frequency of binucleated cells were identified in smokeless tobacco users (SMD = 0.29, with a 95% CI -0.54 to 1.12; *p* = 0.50;  Table 3). Data were heterogeneous (*p* < 0.00001; I2 = 94%;  Table 3).

**Table 3:**
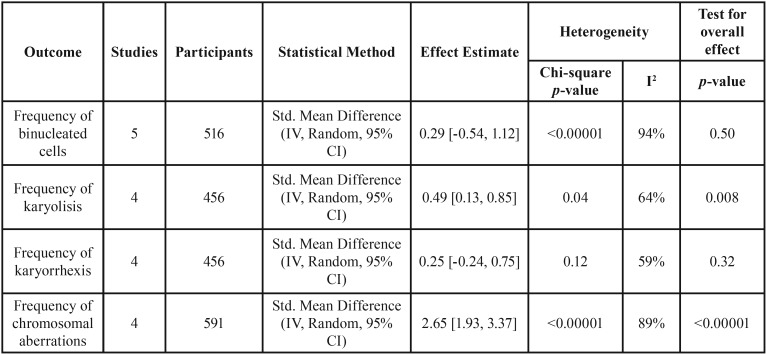
Data and analyses of other outcomes.

-Frequency of karyolisis

This analysis was based in 4 studies (1,8,24,27). A significant higher frequency of karyolisis were identified in smokeless tobacco users (SMD = 0.49; 95%CI 0.13 to 0.85; (*p* = 0.008;  Table 3). Data were heterogeneous (*p* = 0.04; I2 = 64%;  Table 3).

-Frequency of karyorrhexis

This analysis was based only on 2 studies (8,27), because the mean and SD of other 2 studies (1,24) for non-users was zero, so we could not meta-analyze these data. The SMD of the frequency of micronuclei between groups was 0.25, with a 95% CI of -0.24 to 0.75 (*p* = 0.12). Based on these studies, a significant difference between groups were not identified ( Table 3). Data were not heterogeneous (chi-square test, *p* = 0.32; I2 = 59%;  Table 3).

-Frequency of chromosomal aberrations

This analysis was based on 4 studies (11,21,31,32). A significant higher frequency of chromosomal aberrations were identified in smokeless tobacco users (SMD = 2.65; 95%CI 1.93 to 3.37; (*p* < 0.00001;  Table 3). Data were heterogeneous (*p* < 0.00001; I2 = 89%;  Table 3).

-Sensitivity analysis

In order to identify some predictor factors that could be responsible for the high heterogeneity observed in the frequency of micronuclei, we evaluated the impact of some variables on the effect estimate and the heterogeneity. Among them, we investigated the staining method (Feulgen, Papanicolau or Giemsa), the local of smear (cheek, lower lip or buccal vestibule), the quality of studies (strong or moderate), the smokeless tobacco habit time (less or more than 5 years), and the type of smokeless tobacco habit (snuff or chewing).

In all cases, we did not observe significant reduction of the heterogeneity, which remained high in all situations (data not shown). Except from the meta-analysis of binucleated cells, heterogeneity was still present but with no change in the direction of the estimate.

## Discussion

Increased MN frequency and DNA damage were observed in smokeless tobacco users than patients that do not have this habit. Tobacco itself is a very carcinogenic agent (15) as many carcinogens have been identified in smokeless tobacco. Chewing and snuff tobacco generate free radicals that reduce the antioxidant property of saliva and create a pro-oxidant environment in the oral cavity and produce reactive oxygen species due to the presence of specific nitrosamines, which can induce tumors in the oral cavity (3). Various tobacco specific nitrosamines have been found in saliva of smokeless tobacco products consumers (3).

Most of studies’ participants was male, an expected finding since it was demonstrated that rates of consumption of smokeless tobacco have increased significantly amongst specific subgroups of men, particularly young college men (35). This is consistent with the findings of the present systematic review, since the age of the participants ranged from 15 to 80 years, showing that this habit begins when they are still young in some countries. Considering the age difference of study participants, the time of habit also varied greatly, from 1 to 51 years.

Different mixtures were used for snuff and chewing, but most studies evaluated maras powder and betel quid and users of these products constitute a high-risk group for the development of oral malignancies (1,12,13,16,17,28). There is a greater exposure of a very limited mucosal surface when users routinely place these products in the same place each time they consume, and the process continues for years.

In order to collect the cells, the cheek is recommended as the smear site, using a wooden or metal spatula or a cytobrush moistened with water, to gently rub the mucosa. In some cases, oral cells were also collected from the inner side of the lower lip and palate; but it has been already demonstrated that the variability in MN frequency between these areas was minimal compared to controls (36).

The number of counting cells in evaluated studies varied a lot, but most of them counted 1000 cells. A statistical calculation of the ideal number of exfoliated buccal cells to be counted would be required, due to the low frequency of MN in these cells. It is anticipated that more accurate results will be obtained with larger numbers of cells, to a point where the additional precision obtained would not be worth the additional effort (37).

For the evaluation DNA damage in cells, different stains were used for coloring cells. While designing and conducting the study, staining methods themselves may cause significant variations (38). Still, while using a non-DNA specific stain like Giemsa stain, there are inherent chances of counting particles other than MN such as keratohyaline granules and bacteria, which take up the stain, as MN. Giemsa stain produces significant overcount of the mean values of MN when compared to DNA specific stain (22). Papanicolau stain is considering the best staining technique for cytological smears since it provides a polychromatic, transparent staining reaction with crisp nuclear and cytoplasmic features (22). Feulgen stain was recommended as permanent slides can be obtained that can be viewed under both transmitted and/or fluorescent light conditions (38). The data of different staining methods were merged since they evaluated the same outcome. To compensate for this different coloring methods, standardized mean difference instead of mean difference was used to summarize the study results.

MN originate from chromosome fragments or whole chromosomes that lie behind in the anaphase during nuclear division. Some cells, with or without MN, may degenerate into cells with condensed chromatin, fragmented nuclei (karyorrhectic cells), pyknotic nuclei or completely lose their nuclear material (karyolytic cells) (39). In rare cases, cells may be blocked in a binucleated stage or may display nuclear buds (also known as “broken eggs”) (39). All these biomarkers of genomic damage can be observed in the oral cell system and thus provide a more comprehensive assessment of genomic damage. For this reason, we evaluated MN and other biomarkers.

On a sensitivity analysis, we could not reduce the heterogeneity of the meta-analyses by controlling factors such staining method, the local of smear, the quality of studies, the smokeless tobacco habit time and the type of smokeless tobacco. This imply that other factors may have an important role on the high heterogeneity of the results. Some variables such as age, gender, genotype, season, diet, oral hygiene, dental health, life‑style and recreational drugs may affect the MN frequency (40). Large, multicenter, longitudinal studies involving a standard method are needed to affirm the findings of the present systematic review.

## Conclusions

Despite the heterogeneity of studies, the frequency of micronuclei and other DNA damage (frequency of binucleated cells, karyolisis, karyorrhexis and chromosomal aberrations) was significant higher in adults who have smokeless tobacco habits.
